# NUDT22 promotes cancer growth through pyrimidine salvage

**DOI:** 10.1038/s41388-023-02643-4

**Published:** 2023-03-04

**Authors:** Melanie Walter, Florian Mayr, Bishoy M. F. Hanna, Victoria Cookson, Oliver Mortusewicz, Thomas Helleday, Patrick Herr

**Affiliations:** 1 Department of Oncology and Metabolism, University of Sheffield, Sheffield, S10 2RX UK; 2 Science for Life Laboratory, Department of Oncology and Pathology, Karolinska Institute, 171 76 Stockholm, Sweden

**Keywords:** Targeted therapies, DNA replication

## Abstract

The NUDIX hydrolase NUDT22 converts UDP-glucose into glucose-1-phosphate and the pyrimidine nucleotide uridine monophosphate but a biological significance for this biochemical reaction has not yet been established. Glucose-1-phosphate is an important metabolite for energy and biomass production through glycolysis and nucleotides required for DNA replication are produced through energetically expensive de novo or energy-efficient salvage pathways. Here, we describe p53-regulated pyrimidine salvage through NUDT22-dependent hydrolysis of UDP-glucose to maintain cancer cell growth and to prevent replication stress. *NUDT22* expression is consistently elevated in cancer tissues and high *NUDT22* expression correlates with worse survival outcomes in patients indicating an increased dependency of cancer cells to NUDT22. Furthermore, we show that *NUDT22* transcription is induced after inhibition of glycolysis, MYC-mediated oncogenic stress, and DNA damage directly through p53. *NUDT22*-deficient cancer cells suffer from growth retardation, S-phase delay, and slower DNA replication fork speed. Uridine supplementation rescues replication fork progression and alleviates replication stress and DNA damage. Conversely, *NUDT22* deficiency sensitizes cells to de novo pyrimidine synthesis inhibition in vitro and reduces cancer growth in vivo. In conclusion, NUDT22 maintains pyrimidine supply in cancer cells and depletion of NUDT22 leads to genome instability. Targeting NUDT22 therefore has high potential for therapeutic applications in cancer therapy.

## Introduction

Genetic instability in cancer is often caused by oncogene-induced replication stress [1, 2], which is in part a consequence of an inaccurate supply of deoxynucleotides (dNTPs) at replication forks [3, 4]. Targeting the dNTP supply through anti-folates, thymidylate synthetase (TYMS) or ribonucleotide reductase (RNR) inhibitors has remained the backbone for anticancer treatments for over half a century, and provoking replication stress through inhibition of PARP or other DNA repair proteins remains a key area for future cancer therapies [5]. Thus, understanding dNTP synthesis pathways is important for our ability to identify novel cancer vulnerabilities.

We previously reported that the NUDIX family gene *NUDT22* encodes a UDP-glucose hydrolase that converts UDP-glucose to uridine-monophosphate (UMP) and glucose-1-phosphate (G-1-P) [6]. Nudix family proteins were found to have a wide range of substrates [7] and were suggested as anticancer targets [8, 9]. However, phylogenetic sequence analysis revealed NUDT22 as a significant outlier [10], and any biological role of this enzyme has yet to be uncovered.

## Results

### *NUDT22* expression is induced by cellular stress

**Fig. 1:**
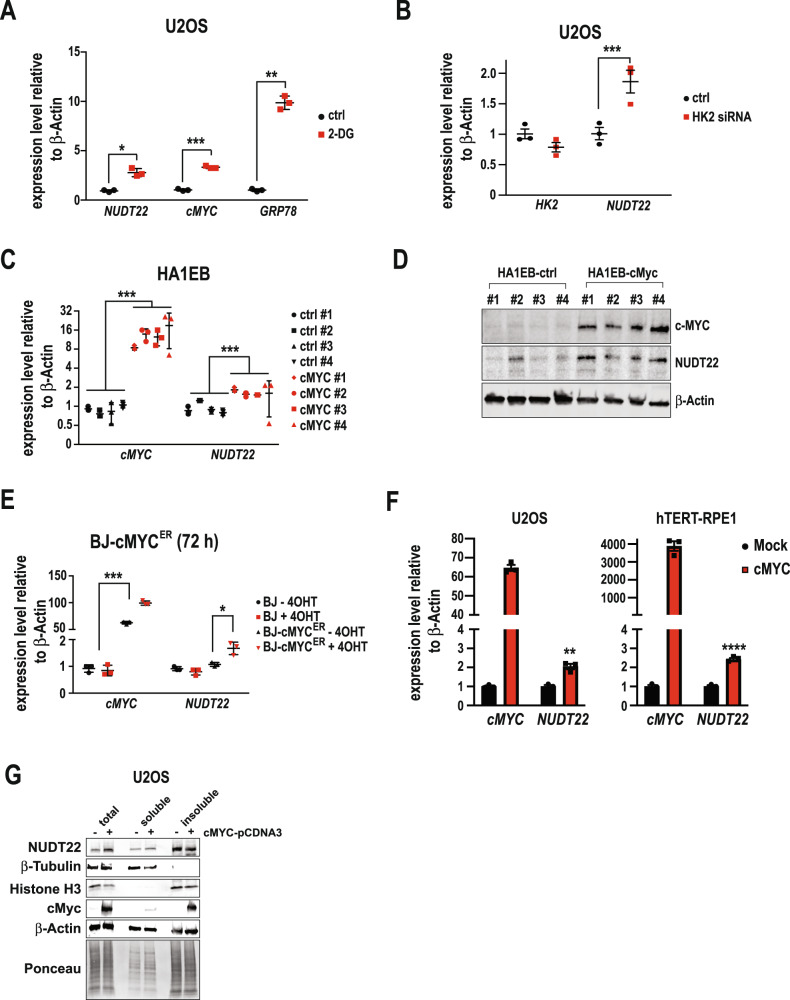
NUDT22 expression is increased in response to stress. **A** Increased *NUDT22* expression after inhibition of HK2 with 2-DG measured by qRT-PCR (*NUDT22* *P* = 0.0254; *cMYC* *P* = 0.0001; GRP78 *P* = 0.0016). *cMYC* and *GRP78* are positive controls. **B** Increased *NUDT22* expression after HK2 depletion with siRNA measured by qRT-PCR (HK2 *P* = 0.006; NUDT22 *P* < 0.001). **C** Elevated *NUDT22* expression in four independent HA1EB-cMYC clones measured by qRT-PCR (*NUDT22* *P* = 2.4*10^−8^; *cMYC* *P* = 7.48 *10^−7^). **D** Western blot of cMYC-overexpressing cells with increased NUDT22 protein levels. **E** cMYC-induced *NUDT22* expression measured by qRT-PCR (*cMYC* *P* = 0.0006; *NUDT22* *P* = 0.0346). **F** Transient cMYC overexpression in U2OS (*NUDT22* *P* = 0.019) and hTERT-RPE1 (*NUDT22* *P* = 0.0002) cells. **G** Western blot of cells fractionated in soluble (cytosolic) and insoluble (nuclei, membranes) of U2OS cells transiently transfected with cMYC. *P* values were calculated by paired *t* test. Data are presented as the mean values with SD and all experiments were repeated at least 3 times.

Both metabolic products of NUDT22 activity, UMP and G-1-P, are generated from UDP-glucose, originating from extracellular glucose. To interrogate the effects of disrupting glucose influx on *NUDT22* expression we blocked the first committed step in glucose metabolism, glucose phosphorylating enzyme hexokinase 2 (HK2)-mediated phosphorylation of glucose to glucose-6-phosphate. Exposure of cells to the HK2 inhibitor 2-deoxy-glucose (2-DG) (Fig. 1A), or RNAi-mediated silencing of *HK2* (HK2^siRNA^) (Fig. 1B) resulted in a significant upregulation of *NUDT22* gene expression, indicating positive feedback to increase the release of G-1-P and UMP from UDP-glucose. Both UMP and G-1-P support the generation of nucleotides, either directly by converting UMP into both pyrimidines dCTP and dTTP or indirectly though the TCA cycle and the pentose phosphate pathway (PPP).

Glucose metabolism is known to be regulated by cMYC, resulting in the activation of genes involved in nucleotide metabolism, glucose uptake, and the serine biosynthesis pathway [11], a critical mechanism for cancer cell survival [12]. Furthermore, cMYC overexpression leads to replication stress [13]. To investigate whether the elevated expression of *NUDT22* after interference with glycolysis was directly regulated through cMYC, we used HA1EB cells (HEK293T cell derivative) with constitutively high [14] (Fig. 1C, D), BJ cells with tamoxifen-induced (4-OHT) short-term cMYC activation [15] (Fig. 1E) and transient cMYC overexpression in U2OS and hTERT-RPE1 cells (Fig. 1F). qRT-PCR analysis revealed that all scenarios led to a significant increase in *NUDT22* expression by qRT-PCR and western blot in the fibroblast cell lines HA1EB, BJ, and hTERT-RPE1 as well as the osteosarcoma U2OS cell line.

UDP-glucose is a cytosolic substrate and NUDT22 subcellular localisation has so far not been confirmed. We used our observation that increased *cMYC* expression leads to elevated *NUDT22* expression to ask whether *cMYC* would influence the subcellular localisation of NUDT22, in particular an increase in the cytosol, to ensure efficient hydrolysis of UDP-glucose. Separation of U2OS cells transiently transfected with cMYC into soluble (cytosolic) and insoluble (nuclei, membranes) fractions followed by western blot analysis confirmed a slight increase in the cytosolic amount of NUDT22 (Fig. 1G). Taken together, *NUDT22* gene expression and protein localisation is influenced by changes in glucose metabolism.

### *NUDT22* is a direct p53 target

Besides its positive role in glucose and nucleotide metabolism, cMYC overexpression was previously shown to induce *p53*, *p21* and senescence [15]. In a largely opposing role to cMYC in glucose metabolism, glycolysis is tightly regulated by p53, controlling the transcription of the glucose transporters *GLUT1* and *GLUT4* and reducing the expression of *HK2* [16] and 6-phosphofrukto-2-kinase 3 (*PFKFB3)*, thereby decreasing glycolysis in favour of the PPP [17]. Nucleotide synthesis, on the other hand, is promoted through expression of the RNR gene *p53R2* directly by p53 [18], and p53 was shown to promote DNA replication and prevent replicative stress [19].

**Fig. 2:**
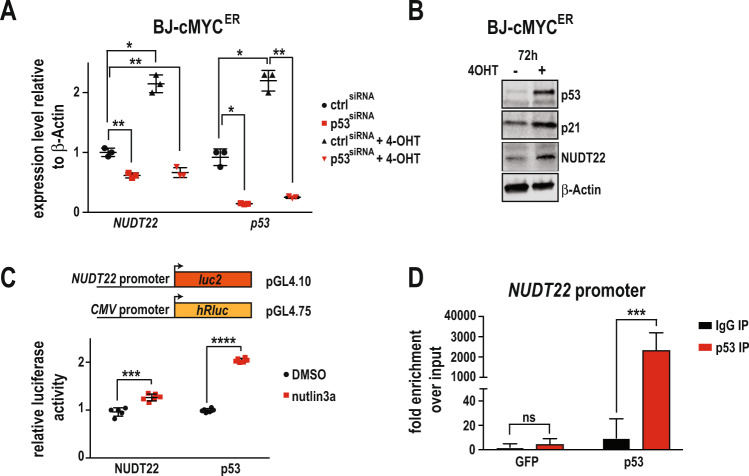
NUDT22 is a p53 target gene. **A** The depletion of *p53* abolished the cMYC-mediated activation of *NUDT22* in BJ-MYC^ER^ cells (*NUDT22* *P* = 0.0086; *P* = 0.0119; *P* = 0.0046; *p53* *P* = 0.012; *P* = 0.0146; *P* = 0.0022), measured by qRT-PCR. **B** Western blot of stabilized p53 and p21 and increased NUDT22 in BJ-MYC^ER^ cells. **C** Relative luciferase levels of the *NUDT22* reporter after stabilization of p53 with Nutlin3a in U2OS cells (*NUDT22* *P* = 0.0003, *p53* *P* = 0.0001). **D** qRT-PCR for the 2 kb CpG 5ʹ region of the *NUDT22* gene after ChIP with a p53(DO1) antibody in U2OS cells. GFP served as a transfection control (P = 0.008). *P* values were calculated by paired *t* test. Data are presented as the mean values with SD and all experiments were repeated at least 3 times.

In silico analysis of the *NUDT22* promoter revealed several putative p53 binding sites (Alggen Promo, V3) [20]. To test whether *NUDT22* expression is directly regulated by cMYC or rather through p53, we depleted *p53* with RNAi *(p53*^*siRNA*^) in cMYC-overexpressing cells. Strikingly, qPCR analysis of the cMYC-induced gene expression of *NUDT22* revealed complete dependency on p53 (Fig. 2A). Although *NUDT22* expression was reduced in the p53 knockdown only condition there was still a significant amount expressed, suggesting that *NUDT22* basal expression must be maintained by factors other than p53. Indeed, NUDT22 protein and mRNA was still detected in the *p53* KO HCT116 cell line as well as in U2OS cells after RNAi mediated silencing of p53 alongside a slight increase in DNA damage (Supplementary Fig. 1A–C). Conversely western blot analysis of *cMYC* overexpression further confirmed stabilized p53, p21 and NUDT22 (Fig. 2B). This is also consistent with the delay in the gene expression of *NUDT22* and *p53* after cMYC activation (Supplementary Fig. 1D–F) and increased *p53* expression after 2-DG exposure or *HK2*^*siRNA*^ (Supplementary Fig. 1G, H). Furthermore, nutlin3a-mediated p53 stabilization [21] led to induction of *NUDT22* gene expression in p53-proficient cells only (Supplementary Fig. 1I) and direct overexpression of *p53* [22] was sufficient to induce the expression of *NUDT22* (Supplementary Fig. 1J).

To further evaluate the effects of Nutlin3a exposure and therefore stabilised p53 protein directly on the activation of the *NUDT22* promoter we generated a *NUDT22*-luciferase reporter construct (*NUDT22*-luc) (Fig. 2C). Analysis of *NUDT22*-luc activation in the dual luciferase assay with a constitutive CMV-luciferase as internal control further confirmed our findings, again indicating direct activation of the *NUDT22* promoter by p53 (Fig. 2C). Finally, we confirmed direct p53 binding in the *NUDT22* promoter region by chromatin immunoprecipitation followed by qRT-PCR using the *p21* promoter region as positive control (Fig. 2D and Supplementary Fig. 2K).

**Fig. 3:**
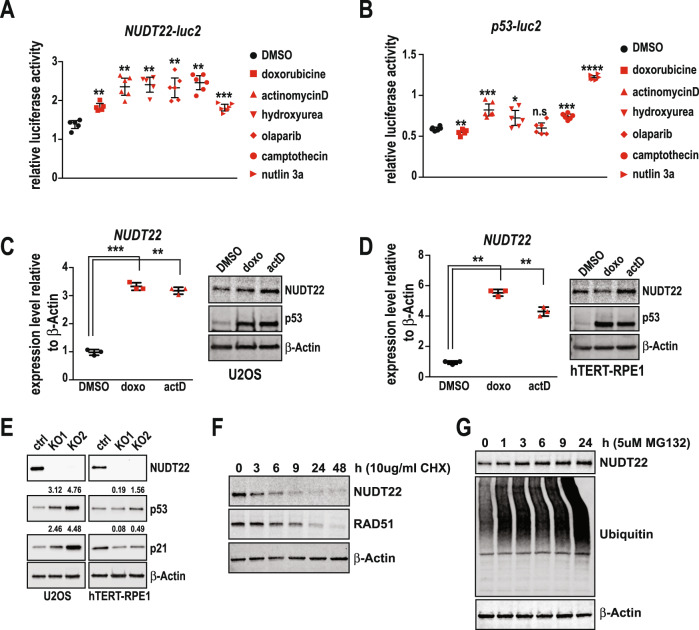
NUDT22 is induced by DNA damage. **A** DNA damaging agents induce transcriptional activation of the *NUDT22* reporter measured by relative luciferase activity. **B** The p53-luciferase reporter served as a control. Drug concentrations: doxorubicine (doxo) 5 μM, actinomycin D (actD) 5 nM, hydroxyurea (HU) 2 mM, olaparib 10 μM, camptothecin 10 μM, nutlin3a 2 μM (*P* values calculated to DMSO control (*NUDT22*): doxo *P* = 0.0094; actD *P* = 0.0036; HU *P* = 0.0088; olaparib *P* = 0.0042; CPT *P* = 0.0021; nutlin3a *P* = 0.0003. (*p53*) doxo *P* = 0.0068; actD *P* = 0.0008; HU *P* = 0.0344; olaparib P = ns; CPT *P* = 0.0003; nutlin3a *p* < 0.0001). **C** U2OS and (**D**) hTERT-RPE1 cells were treated with doxorubicin and actinomycin D for 24 h. *NUDT22* expression was increased in both cell lines as measured by qRT-PCR. This is consistent with the stabilization of p53 measured by western blot (**C**: doxo *P* = 0.0013; actD *P* = 0.0013, **D**: doxo P = 0.0004; actD *P* = 0.0021). **E** Increased p53 stability and activity in U2OS cells after *NUDT22* knockout measured by western blot. Protein level quantification of NUDT22 and p53 is shown in percent normalised to β-Actin and relative to control cells. **F** NUDT22 protein levels were significantly reduced after 6 h of translation inhibition with 10 μg/ml CHX detected by western blot and RAD51 served as a positive control. **G** Western blot of NUDT22 protein levels accumulating after proteasome inhibition with 5 μM MG132. *P* values are calculated by paired *t* test in GraphPad Prism. Data are shown as the mean with SD and all experiments were repeated at least 3 times.

To test whether activation of p53 by different means would also influence *NUDT22* expression we exposed U2OS cells carrying *NUDT22*-luc or *p53*-luc reporters to a spectrum of chemotherapeutic drugs which led to a significant increase in both (Fig. 3A, B). This was again recapitulated after actinomycin D and doxorubicin exposure of U2OS and hTERT-RPE1 cells, correlating with the stabilization of p53 analysed by western blot and increased *NUDT22* gene expression (Fig. 3C, D). These findings demonstrate that induction of cellular stress, in this case by overexpression of the cMYC oncogene or induction of DNA damage, leading to the stabilisation of p53 triggers the transcriptional activation of *NUDT22*.

To interrogate the importance of *NUDT22* in vitro we utilised CRISPR/Cas9 gene editing to generate complete constitutive gene knockouts (*NUDT22* KO). We chose *p53* wildtype osteosarcoma (U2OS) cancer cells and retina pigment epithelial (hTERT-RPE1) fibroblasts as model systems. Importantly, *NUDT22* KO U2OS cells already have increased p53 and p21 levels compared to their respective controls, indicating elevated levels of metabolic stress and suggesting a p53-mediated positive feedback loop regulating *NUDT22* expression. This was, however, not observed in hTERT-RPE1 fibroblasts (Fig. 3E) and similarly also not in the non-cancer MRC5-SV2 and 16HBE14o fibroblasts (Supplementary Fig. 2A, B).

Our findings that *NUDT22* gene expression is induced under conditions of cellular stress in a p53 dependent manner could imply the strict regulation of *NUDT22* to be only present at elevated levels when metabolic demand is high. This prompted us to investigate the stability of NUDT22 protein itself. Inhibition of translation by cycloheximide (CHX) revealed that NUDT22 is rapidly degraded. Already after 6 h of CHX exposure NUDT22 protein levels are hardly detectable (Fig. 3F). Interestingly, this degradation was not delayed when cells were treated with DNA damaging agents that triggered a robust p53 response (Supplementary Fig. 3A, B). Inhibition of the proteasome with MG132 on the other hand led to a continuous increase in NUDT22 protein levels (Fig. 3G). These findings further support a role for NUDT22 in acute situations where nucleotide/energy shortage requires immediate salvage of pyrimidines and G-1-P. In summary, cellular stress such as DNA damage or oncogene activation leads to a direct p53-mediated induction of *NUDT22* expression.

### NUDT22 prevents replication stress

**Fig. 4:**
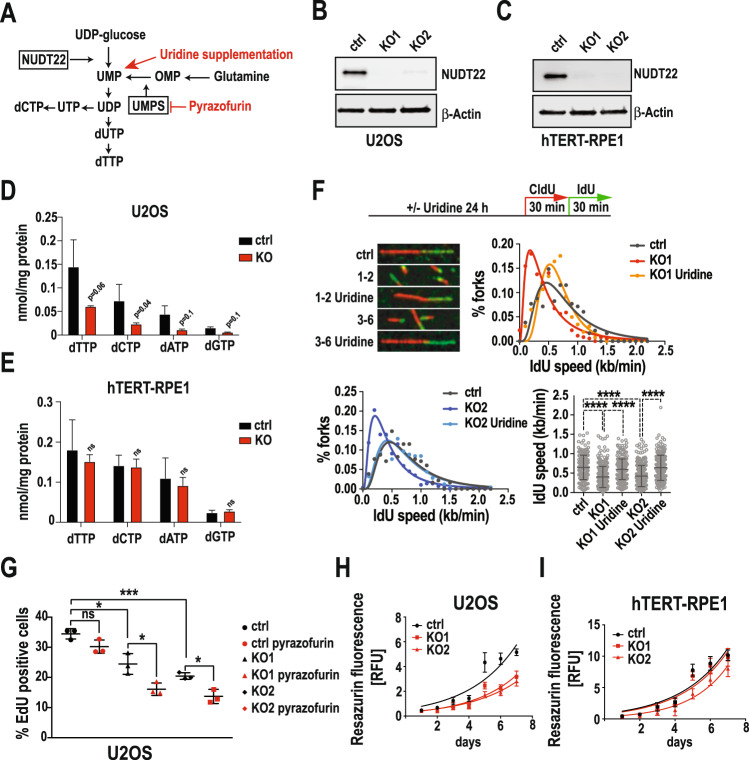
Loss of NUDT22 leads to replication stress. **A** Model of NUDT22 in UDP-glucose hydrolysis as a UMP salvage pathway. **B** U2OS *NUDT22* knockout (KO) cells. Western blot of two independent clones are shown. **C** hTERT-RPE1 *NUDT22* knockout (KO) cells. Western blot of two independent clones are shown. **D** LC-MS nucleotide pool measurement of U2OS and (**E**) hTERT-RPE1 cells. **F** Replication fork speed (IdU incorporation) in ctrl and *NUDT22* KO cells. **G** Quantification of the percentage of EdU-positive cells by high content microscopy (ctrl::KO1 *P* = 0.0109; ctrl::KO2 *P* = 0.0003; KO1 DMSO::KO1 pyrazofurin *P* = 0.0227; KO2 DMSO::KO2 pyrazofurin *P* = 0.0109). *P* values were calculated by unpaired *t* test. Errors as the mean with SD. **H** Growth rates of U2OS and (**I**) hTERT-RPE1 cells determined by resazurin fluorescence. All experiments were repeated at least 3 times.

Our biochemical data show that NUDT22 generates the pyrimidine synthesis precursor UMP from UDP-glucose [6] (Fig. 4A). We therefore aimed to determine the biological significance of NUDT22 for dNTP production and DNA replication in cells. To this end we compared the cellular role of NUDT22 in U2OS cancer and hTERT-RPE1 fibroblast *NUDT22* KO cell lines (Fig. 4B, C). dNTP pool measurements by LC-MS confirmed that *NUDT22* KO U2OS cells had reduced levels of all 4 dNTPs but only marginal changes were observed in hTERT-RPE1 cells (Fig. 4D, E). The reduction in intracellular dNTPs in *NUDT22* KO U2OS cells was reflected in a significantly reduced DNA replication fork speed. To test whether the reduced replication fork speed in *NUDT22* KO cells is a consequence of attenuated UMP production, we supplemented *NUDT22* KO cells with uridine. Supporting our hypothesis, uridine supplementation completely restored replication fork speed, demonstrating that the lack of NUDT22-dependent hydrolysis of UDP-glucose to UMP is required for normal replication fork progression (Fig. 4F and Supplementary Fig. 4A). The attenuated replication fork speed is further reflected by reduced EdU incorporation during S-phase (Fig. 4G and Supplementary Fig. 4B) and a generally slower proliferation speed in *NUDT22* KO U2OS but not hTERT-RPE1 cells (Fig. 4H, I).

These findings prompted us to hypothesize that NUDT22 controls a novel pyrimidine salvage pathway and might therefore synergise with de novo pyrimidine synthesis (Fig. 4A). A key enzyme in the de novo synthesis of pyrimidines from glutamine is uridine monophosphate synthetase (UMPS), which converts orotidine 5’-phosphate to UMP [23]. Inhibition of UMPS with pyrazofurin [24] caused a further reduction in EdU incorporation in *NUDT22* KO cells progressing through S-phase (Fig. 4G and Supplementary Fig. 4B), demonstrating that NUDT22 is involved in a complementary pathway for UMP generation.

**Fig. 5:**
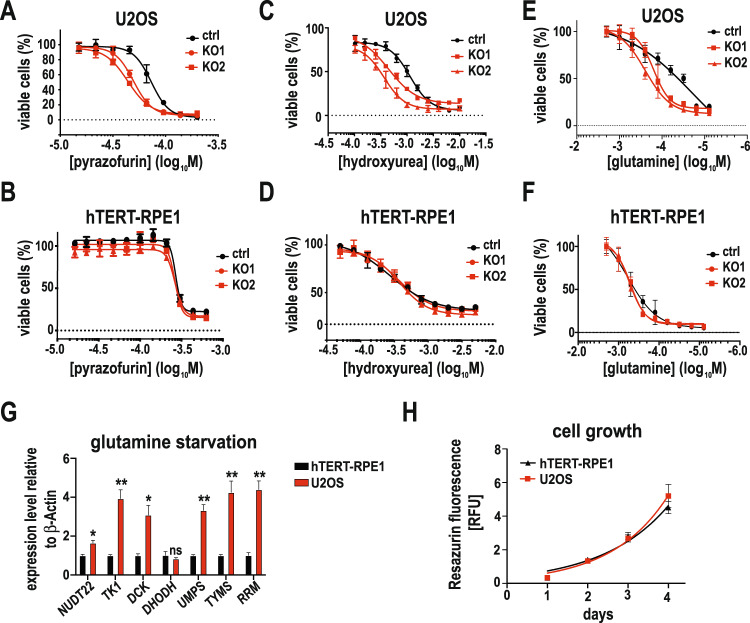
Loss of NUDT22 potentiates inhibition of nucleotide metabolism. Dose response curves of ctrl and *NUDT22* KO U2OS cells exposed to (**A**) pyrazofurin, (**C**) hydroxyurea and (**E**) after glutamine starvation. Dose response curves of ctrl and *NUDT22* KO hTERT-RPE1 cells exposed to (**B**) pyrazofurin, (**D**) hydroxyurea and (**F**) after glutamine starvation. **G** Expression levels of pyrimidine synthesis genes after 24 h of glutamine starvation relative to β-actin measured by qRT-PCR. Statistical analysis between hTERT-RPE1 and U2OS cells (*NUDT22* *P* = 0.0135; *TK1* *P* = 0.0071; *DCK* *P* = 0.0132; *DHODH* P = ns; *UMPS* *P* = 0.004; *TYMS* *P* = 0.01; *RNR* *P* = 0.0026). *P* values were calculated by paired *t* test. Data are presented as the mean values with SD. **H** Growth rate comparison between U2OS and hTERT-RPE1 cells. All experiments were repeated at least 3 times.

These findings further suggest that *NUDT22* KO cells might be especially sensitive to pyrazofurin. Exposure of *NUDT22* KO cells and their respective controls to pyrazofurin in dose-response survival assays revealed a clear sensitization in U2OS but not hTERT-RPE1 cells (Fig. 5A, B). Similar combinatorial effects were observed after inhibition of RNR with hydroxyurea (HU) (Fig. 5C, D) or by starving cells from the de novo pyrimidine precursor glutamine (Fig. 5E, F), phenocopying the effect of pyrazofurin exposure and further supporting a shortage in nucleotide supply in *NUDT22*-deficient cells. The difference in sensitivity between the U2OS cancer and hTERT-RPE1 fibroblast cell lines prompted us to also test the sensitivity in additional fibroblast cell lines MRC5-SV2 and 16HBE14o, both of which did not show a differential response when exposed to the pyrimidine synthesis inhibitors pyrazofurin or brequinar nor RNR inhibition with HU (Supplementary Fig. 5A).

Interestingly, glutamine starvation led to the upregulation of key pyrimidine biosynthesis enzymes and *NUDT22*, especially in U2OS cells further supporting an important role for NUDT22 in the maintenance of pyrimidine nucleotide levels (Fig. 5G).

Besides inhibiting pyrimidine synthesis through UMPS, pyrazofurin was shown to also negatively affect purine biosynthesis [25]. We therefore tested whether the observed combinatorial effects are specifically related to a deficiency in pyrimidine synthesis. Inhibition of purine synthesis with mycophenolic acid (MPA) or 6-mercaptopurine (6-MP) did not increase the sensitivity of *NUDT22* KO U2OS or hTERT-RPE1 cells, further supporting a specific role for NUDT22 in pyrimidine biosynthesis (Supplementary Fig. 4C, D). Importantly the difference in response between U2OS and hTERT-RPE1 cells is not attributable to differential proliferation rates of the two cell lines (Fig. 5H).

**Fig. 6:**
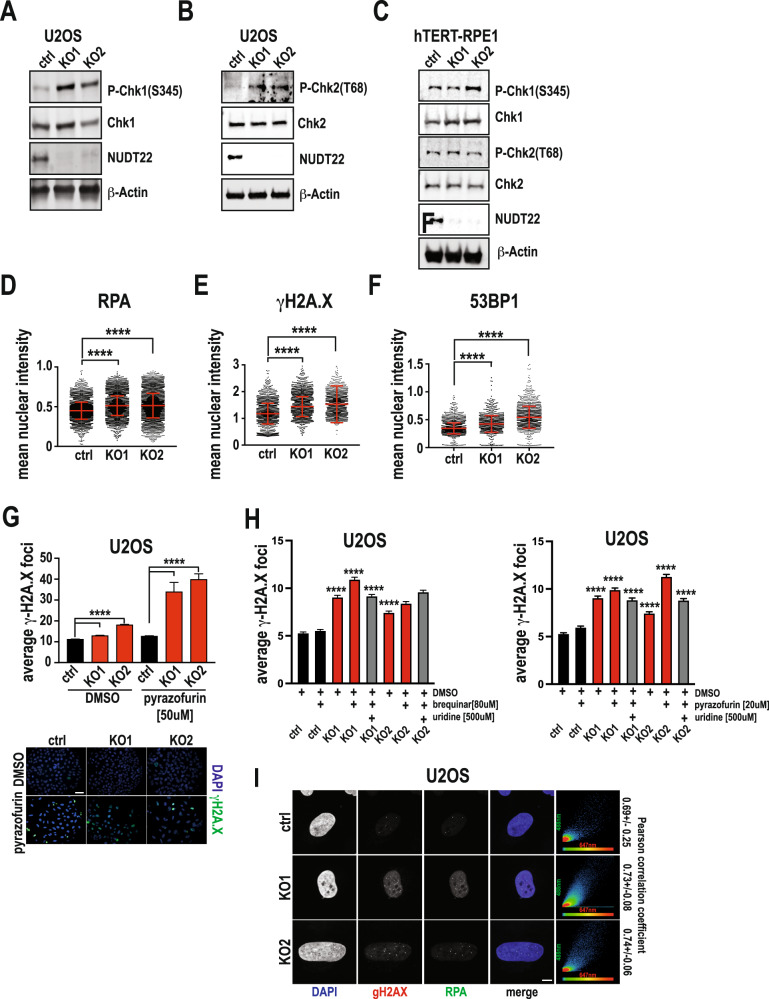
Loss of NUDT22 activates DNA damage response. **A**, **B** Cell cycle checkpoint activation in *NUDT22* KO U2OS cells measured by western blot. **C** No significant cell cycle checkpoint activation in *NUDT22* KO hTERT-RPE1 cells measured by western blot. **D** Increased single stranded DNA (nuclear RPA intensity) and DNA damage (nuclear γH2A.X (**E**) and 53BP1 (**F**) intensity) quantified by high content immunofluorescence microscopy. **G** Quantification of γH2A.X DNA damage foci in ctrl and *NUDT22* KO U2OS cells with and without pyrazofurin by high content immunofluorescence microscopy (*P* < 0.0001). **H** Uridine supplementation reverses the DNA damage in *NUDT22* KO U2OS cells exposed to brequinar or pyrazofurin. **I** Confocal microscopy reveals strong colocalization of nuclear RPA and γH2A.X in *NUDT22* KO U2OS cells (scale bar:10 μM). *P* values were calculated by the Mann–Whitney test. Data are presented as the mean values with SEM. All experiments were repeated at least 3 times.

Nucleotide deficiency and reduced replication fork progression are often associated with replication stress. Consistent with the reduction in pyrimidine synthesis, we observed increased cell cycle checkpoint activation in *NUDT22* KO U2OS but not hTERT-RPE1 cells (Fig. 6A–C) and an increase in markers for replication stress (RPA) and DNA damage (γH2A.X, 53BP1) (Fig. 6D–F). DNA damage (γH2A.X) was further increased when combined with low doses of pyrazofurin (Fig. 6G) and brequinar (Fig. 6H), which was reversed by the addition of uridine (Fig. 6H). Again, further strengthening the hypothesis that the dependency on NUDT22 appears to be enhanced in cancer cells compared to normal fibroblasts we did not detect significantly increased DNA damage (γH2A.X) or replication stress (RPA) in MRC5-SV2 or 16HBE14o cells and this was also not enhanced by additional pyrimidine synthesis inhibition (Supplementary Fig. 5B). Importantly both markers for replication stress (RPA) and DNA damage (γH2A.X) colocalised in *NUDT22* KO U2OS cells (Fig. 6I).

Previous work analysing gene expression levels across the cell cycle in U2OS-Fucci cells suggest a potential cell cycle regulation of *NUDT22* with a slight increase in expression in S phase of the cell cycle [26] (Supplementary Fig. 6A). To further address this, we performed a double thymidine cell cycle synchronisation experiment and detected only slightly increased NUDT22 protein levels after G1/S boundary release while progressing towards G2/M followed by a decrease in abundance in U2OS cells but not hTERT-RPE1 cells (Supplementary Fig. 6B). Again, these findings support a role for NUDT22 in providing additional pyrimidine nucleotides to support DNA replication during S-phase.

### NUDT22 deficiency reduces cancer growth

**Fig. 7:**
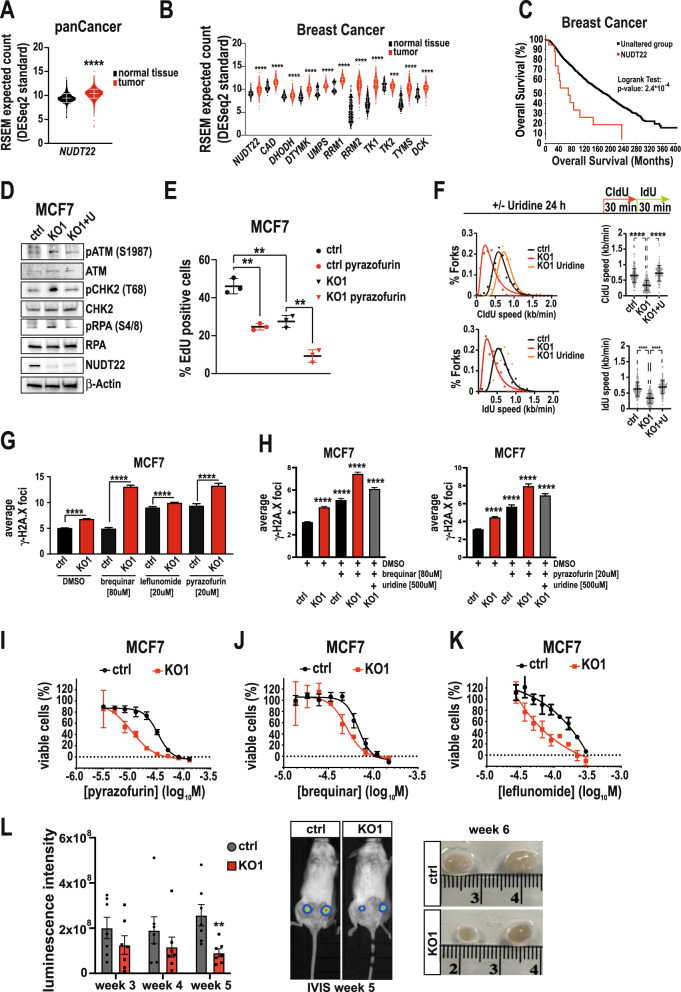
NUDT22 expression is increased in cancer and loss of NUDT22 reduces cancer growth in vivo. **A** RSEM expected count analysis of *NUDT22* expression in the panCancer TCGA and GTEx datasets (*P* < 0.0001; Mann–Whitney test, mean with SD). **B** RSEM expected count analysis of pyrimidine metabolism gene expression in breast cancer TCGA and GTEx datasets (*P* < 0.0001; Mann–Whitney test, mean with SD). **C** Overall survival (OS) of breast cancer patients with *NUDT22* gene alterations (TCGA PanCancer). Altered group is defined as patients with at least one alteration in the *NUDT22* gene. **D** Cell cycle checkpoint activation of ctrl and *NUDT22* KO MCF7 cells was rescued by uridine supplementation detected by western blot. **E** *NUDT22* KO MCF7 cells have reduced EdU incorporation, which is further reduced by pyrazofurin exposure quantified by high content microscopy (mean with SD). **F** Reduced replication fork speed in *NUDT22* KO MCF7 cells can be rescued by uridine supplementation (mean with SD). **G** *NUDT22* KO MCF7 cells have increased gH2A.X foci formation, which was further increased with brequinar, leflunomide and pyrazofurin quantified by high content immunofluorescence microscopy (*P* < 0.0001). **H** uridine supplementation rescues DNA damage caused by NUDT22KO (*P* < 0.0001) (mean with SEM). *P* values were calculated by the Mann–Whitney *t* test. **I** Dose-response curves of ctrl and *NUDT22* KO MCF7 cells treated with pyrazofurin, (**J**) brequinar and (**K**) leflunomide**. L** In vivo mammary cancer xenograft model with ctrl and *NUDT22* KO MCF7 cells. *Luc2*^+^ cells were injected into mammary fat pads and imaged weekly by IVIS imaging (*P* < 0.0023; Mann–Whitney test, mean with SEM). All experiments were repeated at least 3 times.

To better understand the overall significance of NUDT22 in cancer, we interrogated the TCGA and GTEx databases for differential gene expression of *NUDT22* and genes involved in pyrimidine biosynthesis. Pan-cancer analysis clearly indicated increased *NUDT22* expression levels in cancer tissue compared to normal tissue (Fig. 7A). Consistent with our previous findings that *NUDT22* expression is induced by cMYC and regulated by p53, overall survival of patients with high *NUDT22* and *cMYC* expression has a significantly worse outcome in several types of cancer (Supplementary Fig. 7A, B) and *MYC* and *NUDT22* expression correlates in the CCLE dataset of 1070 cancer cell lines (Supplementary Fig. 7C). To investigate the role of p53 status in these cancers we separated samples into 3 groups based on the p53 mutational status (no variant/silent, mutation, hot-spot mutation). Again, *NUDT22* expression is elevated in tumour over normal tissue and is not significantly influenced by *p53* mutations (Supplementary Fig. 7D). In fact, the correlation between *NUDT22* and *MYC* in normal tissue, tumour and tumour with *p53* mutations is throughout positive with the strongest correlation in tumours with p53 hot-spot mutations (Supplementary Fig. 7E). Taken our experimental data in consideration these findings further support a positive regulation of *NUDT22* by p53 but also again suggest additional transcriptional factors that drive *NUDT22* expression under different conditions also in the absence of wildtype p53 (Fig. 2A and Supplementary Fig. 1A–C).

The role of other members of the NUDIX protein superfamily was previously described in breast cancer with NUDT2 and NUDT5 as best explored examples [27–29]. Our observed elevated *NUDT22* expression we observed in other cancer types was further reinforced in breast cancer, with all major pyrimidine metabolism enzymes following the same trend (Fig. 7B). This correlates with the previously described role of other members of the NUDIX protein superfamily in breast cancer [30]. In addition, breast cancer patients [31] with *NUDT22* alterations (Fig. 7C) or high NUDT22 expression levels (Supplementary Fig. 8A) have a worse prognosis in overall survival.

To test whether our results in U2OS cells translate in breast cancer cells we generated *NUDT22* KO MCF7 cells. Similar to *NUDT22* KO U2OS cells, *NUDT22* KO MCF7 cells exhibited an increase in the phosphorylation of replication stress markers, which was rescued by uridine supplementation (Fig. 7D). *NUDT22* KO MCF7 cells also had reduced growth rates (Supplementary Fig. 8B) and reduced EdU incorporation compared to control cells, which was further exaggerated by UMPS inhibition with pyrazofurin (Fig. 7E and Supplementary Fig. 8C). The reduced replication fork speed observed in U2OS cells was again recapitulated in MCF7 cells, which was rescued by uridine supplementation (Fig. 7F and Supplementary Fig. 8D). *NUDT22* KO MCF7 cells have increased DNA damage (γH2A.X), further exaggerated by low doses of dihydroorotate dehydrogenase (DHODH) (brequinar, leflunomide) and UMPS (pyrazofurin) inhibitors (Fig. 7G), which can be rescued by uridine supplementation (Fig. 7H). *NUDT22* KO MCF7 cells were, similar to U2OS cells, significantly sensitized to inhibition of UMPS and DHODH (Fig. 7I–K).

To transfer our findings into an orthotopic mouse breast cancer in vivo xenograft model, we injected engineered *luc2 NUDT22* KO MCF7 cells into female NOD/SCID mice and monitored tumour growth by IVIS imaging in live animals. Recapitulating our data on cultured cells, *NUDT22* KO MCF7 cells grew significantly slower than the control cells over the course of the experiment (Fig. 7L). These findings underscore the significance of NUDT22 for cancer cell growth in vivo and provide further support for the potential of targeting NUDT22 in cancer.

## Discussion

Here, we present evidence that NUDT22 is a previously unknown important regulator of a cellular salvage pathway with special significance in cancer. We show that cancer tissues have elevated levels of *NUDT22* and that *NUDT22* expression is directly regulated by p53 following metabolic stress, cMYC overexpression, and DNA damage. *NUDT22*-deficient cancer cells have diminished nucleotide pools and display hallmarks of replication stress, such as reduced replication fork speed, delayed S-phase progression, cell cycle checkpoint activation, increased DNA damage and single-stranded DNA. Furthermore, *NUDT22* expression was found to be increased during S-phase of the cell cycle, which is consistent with a supportive role in nucleotide metabolism and DNA replication [26].

Nucleotide salvage through recycling from intermediates in the degradative pathway is an energy-efficient way to generate nucleotides. While pyrimidines have been shown to be re-phosphorylated inside the cell through DCK and TK1 [32], no true pyrimidine salvage pathway has been described to date that resembles the well-known purine salvage pathway around the *HPRT* gene that has been exploited extensively therapeutically and as a biological tool [33]. Co-targeting of pyrimidine salvage and de novo synthesis for cancer therapy has recently received renewed attention [34], and our data suggest that targeting pyrimidine de novo synthesis combined with NUDT22 inhibition might be an interesting novel therapeutic approach in the future. Our data that glutamine starvation synergizes with *NUDT22* KO in U2OS cells (Fig. 3F) therefore further supports this idea. UDP-glucose, the substrate for NUDT22, has been linked to metastatic progression of lung cancer cells by directly interfering with translation of the EMT-promoting gene *SNAI1* [35]. Inhibition/deletion of NUDT22 could therefore potentially suppress metastatic potential and simultaneously increase DNA damage burden in cancer cells by reducing UMP and keeping UDP-glucose levels high.

There seems to be a distinct difference in the level of dependence on NUDT22 in different cell types. We consistently observed much more severe effects in cancer cell lines (U2OS and MCF7) than in fibroblast cell lines (hTERT-RPE1, MRC-SV2, 16HBE14o). All 5 cell lines are p53 wildtype which allows us to exclude a potential bias based on the level of gene regulation. Contrary to the epithelial cancer cells which have undergone transformation in the patient before isolation, the fibroblast cell lines have been immortalised to allow their prolonged cell culture. It is tempting to hypothesize that the observed differences in cancer cell lines may be due to an increased metabolic demand in cancer, which is also suggested by the increased *NUDT22* expression in cancer tissues but needs to be further investigated. Although the majority of phenotypes in *NUDT22* KO could be rescued by uridine supplementation and are clearly directly attributable to UMP, and therefore pyrimidine, deficiency, the significance of G-1-P generated by NUDT22 for the maintenance of cellular growth in fast proliferating cancer cells needs to be addressed. The attack on nucleotide synthesis through a complementary salvage pathway, which cancer cells seem to be more reliant on, might prove advantageous over currently used nucleoside synthesis inhibitors. Finally, our in vivo findings provide a clear rationale for the preclinical translation of targeting NUDT22 in cancer.

## Materials and methods

### Antibodies

NUDT22 (H-9; sc-515491, Santa Cruz Biotechnology, Dallas, TX, US), β-Actin (ab8227, Abcam, Dallas, TX, USA), p53 (DO-1; sc-126), cMyc (C-33; sc-42), P21 (H-164; sc-756), γH2A.X (Millipore 05–636, Merck Life Science, UK Limited, Gillingham, UK), RPA (cs 2208, Cell Signalling Technology, Danvers, MA, USA), 53BP1 (ab36823), pCHK2 (cs2661S), CHK2 (cs3440S), pCHK1 (cs133D3), CHK1 (cs2G1D5), P-RPA (A300–245A, Bethyl Laboratories,Inc., Montgomery, TX, USA), GFP (ab290), ATM (11G12, sc-53173), P-ATM (SAB #12701, SAB Signalway Antibody, Greenbelt, Maryland, USA), CCNB1 (4138, Cell Signalling Technology), Alexa Flour-488 (Thermo Fisher Scientific, Paisly, UK), Alexa-Fluor-647, DAPI (Thermo Fisher Scientific), mouse & rabbit IRDye conjugated ab 680/800 (Licor Biosciences, Lincoln, Nebraska, USA), mouse & rabbit Starbright conjugated ab 520/700 (Biorad, Watford, UK), mouse-hrp (31430, Thermo Fisher Scientific), rat-hrp (A9037, Sigma Aldrich) & rabbit-hrp (ab6721, Abcam).

### Cell culture

All cells were grown at 37 °C containing 5% CO_2_, in a humidified incubator. U2OS and hTERT-RPE1 cells were grown in DMEM Glutamax with 10% FBS, penicillin (60–100 μg/ml), and streptomycin (100 μg/ml). HA1EB were grown in DMEM with 10% FBS, penicillin (60–100 μg/ml), streptomycin (100 μg/ml), BJ-MYC^ER^ in DMEM/F12 without phenol red with 10% FBS, penicillin (60–100 μg/ml), streptomycin (100 μg/ml) and HCT116 in McCoy’s 5a with 10% FBS, penicillin (60–100 μg/ml), and streptomycin (100 μg/ml). MCF7 cells were grown in RPMI with 10% FBS, penicillin (60–100 μg/ml), and streptomycin (100 μg/ml). MRC5-SV2 andn16HBE14o cells were grown in MEM with 10% FBS, penicillin (60–100 μg/ml), and streptomycin (100 μg/ml) (Thermo Fisher Scientific). All cell lines were regularly checked for Mycoplasma contamination (MycoAlert, Lonza, Basel, Switzerland). All cell lines were validated using short-tandem repeat (STR) profiling. For glutamine starvation cells were washed twice in warm PBS, and DMEM containing 10% FBS without glutamine was added for the indicated times.

### Immunofluorescence and microscopy

Immunostaining was performed according to standard protocols in 96 well imaging plates and high-content imaging was performed with an ImageXpress XLS (Molecular Devices, San Jose, CA, USA) or a Celldiscoverer7 (Zeiss, Jena, Germany). Confocal microscopy was performed on a LSM980 Airyscan2 confocal laser scanning microscope (Zeiss, Jena, Germany) with a Plan-Apochromat 63x objective and 3 times digital zoom. The data were analysed with CellProfiler-3.0.0 or Zen blue (Zeiss). For quantitative DNA damage foci analysis, >500 nuclei per condition were analysed. For colocalization analysis single staining was used to determine the respective thresholds and Zen blue software (Zeiss) was used to calculate the Pearson correlation coefficient.

### Molecular cloning and plasmids

pIRES2-EGFP-p53 WT was a gift from Dylan Taatjes (Addgene plasmid # 49242) [22]. The *NUDT22* reporter was cloned by PCR amplification of the genomic region GRCh38:11:64224628:64226607 and ligated into pGL4.10 (Promega, Southampton, UK). The transfection control was CMV-driven control luciferase (pGL4.75-CMV-hRluc). PG13-luc (wt p53 binding sites) was a gift from Bert Vogelstein (Addgene plasmid # 16442) [36]. sgRNA for *NUDT22* knockout in U2OS and hTERT-RPE1 cells was cloned into pX330-U6-Chimeric_BB-CBh-hSpCas9, a gift from Feng Zhang (Addgene plasmid # 42230) [37]. The pCDNA3-cMYC plasmid was gift from Wafik El-Deiry (Addgene plasmid #16011) [38].

### Gene editing

For *NUDT22* gene knockout in U2OS cells the following sgRNA sequences were used: KO1: 5ʹ-AUCCUCUACAACCGGGUUCAGGG-3ʹ; KO2:5ʹ-ACUUUAUUCUUGGAUUCCGUUGG-3ʹ.

For *NUDT22* gene knockout in hTERT-RPE1 cells the following sgRNA sequences were used KO1: 5ʹ-AUCCUCUACAACCGGGUUCAGGG-3ʹ; KO2:5ʹ-GUCCCACUGGAGCGGCCCUAGGG-3ʹ. Transfected cells were selected with puromycin. Genomic DNA from individual knockout clones was amplified with primers flanking the sgRNA recognition sites 5ʹ-CGAGTCTACAGGAATCTTCTTTGTGG-3ʹ and 5ʹ-CCAAGTCACTTGTCCTGCC-3ʹ. The same primers were used for sequencing. For *NUDT22* gene knockout in MCF7 cells, the following sgRNAs were used: 5ʹ-CCGGCUAAAGGCCCAACCC-3ʹ (Sigma HSPD0000120767) for MCF7 KO1. Cells were transfected with Cas9-GFP (Sigma) and selected by FACS for the top 10% GFP + cell population. For MCF7 KO2 sgRNA 5ʹ-GACAAGGAAGUCAUCGGCUG-3ʹ was used (Synthego, Menlo Park, CA, USA). For *NUDT22* gene knockout in MRC5-SV2 and 16HBE14o cells, the following sgRNA was used: 5ʹ-ACUUCCUACCGAGACUUCCU-3ʹ (Synthego, Menlo Park, CA, USA). gDNA from positive clones was extracted and sequenced using the following primers: 5ʹ-ATCCTGAGGTGACCTTGCT-3ʹ, 5ʹ-ACTAGCCACAGCCGATGA-3ʹ and 5ʹ-CCCGTTCAGACCATGGATCC-3ʹ. Non-targeting sgRNA and the identical selection process was used for the respective control cells.

### Dual Luciferase assay

For the Dual-Luciferase® Assay (Promega), U2OS cells were seeded and transfected on a 10 cm dish. Transfection was conducted using jetPEI® (Polyplus, Biopark, Illkirch, France) with a total amount of 5 μg DNA comprised of 9 parts *NUDT22-luc2*-pGL4.10 and 1 part *CMV-hRluc*-pGL4.75. Cells were reseeded on a 96-well plate after 24 h. For analysis cells were washed with PBS. Then, 20 µl of Passive Lysis Buffer was added, and the plate was incubated for 15 min at room temperature on a rocking device. Then, 15 μl of the cell lysate of each well was transferred onto an opti-96-well plate, and the plate was read with the dual luciferase setting on a Hidex Sense plate reader (Hidex, Turku, Finland). Experiment was repeated at least 3 times with 6 technical replicates per experiment.

### Cell transfection

Cells were seeded in 12-well plates, and siRNA pools (ON-Target plus SMARTpool, Dharmacon) were transfected to a final concentration of 5 nM according to the manufacturer’s protocol (INTERFERin, Polyplus). *p53* (L-003329-00-0005), *HK2* (L-006735-00-0005). AllStars negative control siRNA (Qiagen, Hilden, Germany) was used as a control siRNA. Plasmid DNA was transfected with jetPEI® (Polyplus, Biopark, Illkirch, France) according to manufacturer’s protocol.

### Drug exposure

Drugs were spotted using a Tecan D300e dispenser (Tecan, Mannedorf, Switzerland) at the following concentrations with log2 dilution series. pyrazofurin (SML1502, Sigma-Aldrich) (200 μM–5 μM for U2OS and hTERT-RPE1; 200 μM–2 μM for MCF7), MPA (M5255, Sigma-Aldrich) (100 μM–0.05 μM), 6MP (38171, Merck) (100 μM–0.05 μM), glutamine starvation (2 mM-7.8 μM), brequinar (5.08321 Sigma-Aldrich) (200 μM–10 μM), and leflunomide (L5025, Sigma-Aldrich) (400 μM–10 μM), hydoxyurea (H8627, Sigma-Aldrich) (5 mM–0.05 mM). Five hundred cells per well were seeded on 384-well cell culture plates, and viability was assessed with a resazurin assay and read on a Hidex Sense or Molecular Devices ID5 plate reader after 4 days in technical triplicates. Uridine (U3750, Sigma-Aldrich) rescue was performed at a concentration of 500 μM for 24 h. 2-DG (10 mM) was added to cultured cells for 48 h. Doxorubicine (D1515, Sigma-Aldrich), actinomycin D (A1410, Sigma-Aldrich), olaparib (AZD2281, Selleckchem), camptothecin (C9911, Sigma-Aldrich), nutlin3a (SML0580, Sigma-Aldrich).

### Western blotting

Western blotting was carried out following standard protocols with Bio-Rad SDS gradient gels and a Trans-Blot Turbo transfer system (Bio-Rad). Cells were lysed in RIPA buffer for 20 min on ice in the presence of protease inhibitor cocktail (Roche, Basel, Switzerland) followed by sonication with a needle sonicator (Hielscher UP100H (Teltow, Germany); 70% amplitude; 0.7 cycles; 3 cycles). Images were taken with a LI-COR Odyssey FC or Biorad Chemidoc MP. Experiments were repeated at least 3 times.

### RNA extraction and quantitative RT-PCR

Total mRNA was isolated from cells with the Direct-zol RNA Mini Prep kit (Zymo Research, Irvine, CA, USA) or ReliaPrep (Promega) according to the manufacturer’s instructions, and cDNA was generated with the QuantiTect Reverse Transcription kit (Qiagen) or iScript (Bio-Rad). qRT-PCR was performed using SYBR Green (Invitrogen/Life Technologies; Bio-Rad) and the reactions were performed on a Rotor-Gene Q (Qiagen) and CFX96 (Bio-Rad) qRT-PCR machine. β-Actin was used as a normalization control. Relative gene expression changes were calculated using the ΔΔCt method. Primers used are: *NUDT22* (5ʹ-GGCAGCTGGTGGTACATGA-3ʹ; 5ʹ-GTCTCATTTCGGGCGATG-3ʹ), *β -Actin* (5ʹ-CCTGGCACCCAGCACAAT-3ʹ; 5ʹ-GGGCCGGACTCGTCATACT-3ʹ), *cMYC* (5ʹ-TCGGATTCTCTGCTCTCCT-3ʹ; 5ʹ-CCTCATCTTCTTGTTCCTCCTC-3ʹ), *p53* (5ʹ-CTTTCCACGACGGTGACA-3ʹ; 5ʹ-TCCTCCATGGCAGTGACC-3ʹ), *CCNE* (5ʹ- CTCCAGGAAGAGGAAGGCAA-3ʹ; 5ʹ-TCGATTTTGGCCATTTCTTCA-3ʹ) [39], *GRP78* (5ʹ-CATCAAGTTCTTGCCGTTCA-3ʹ; 5ʹ-TCTTCAGGAGCAAATGTCTTTGT-3ʹ), *HK2* (5ʹ-TCCCCTGCCACCAGACTA-3ʹ; 5ʹ-TGGACTTGAATCCCTTGGTC-3ʹ), *TK1* (5ʹ- CAGCTTCTGCACACATGACC-3ʹ; 5ʹ-CGTCGATGCCTATGACAGC-3ʹ), *DCK* (5ʹ- ATATGAAAGTCTGGTTGAAAAGGTC-3ʹ; 5ʹ-AAAGCTGAAGTATCTGGAACCATT-3ʹ), *DHODH* (5ʹ-GCGTGGAGACACCTGAAAA-3ʹ; 5ʹ-TCAGGTAGGAGGCGAAGAGA-3ʹ), *UMPS* (5ʹ-GCATGAAACCAGAATTTCTTCAC-3ʹ; 5ʹ-ACTGTTGGCCAAGATTATCTCC-3ʹ), *TYMS* (5ʹ-CCCAGTTTATGGCTTCCAGT-3ʹ; 5ʹ- GCAGTTGGTCAACTCCCTGT-3ʹ), *RNR* (5ʹ-TGGACCTCTCCAAGGACATT-3ʹ; 5ʹ-GGCTAAATCGCTCCACCA-3ʹ). Experiments were repeated at least 3 times and performed in technical triplicates.

### ChIP-qPCR

U2OS cells were transfected with plasmids encoding for GFP alone or p53-IRES-GFP. After 24 h, the cells were processed for ChIP according to [40]. qPCR was performed as described previously with primers for *P21* [41] and the *NUDT22* promoter region fwd: 5'-CCAGACTTGCCCAAGGTC-3'; rev: 5'-CCATGTCCCCCAAACC-3'. Fold enrichment was calculated over the input control and relative to the IgG mock IP.

### DNA Fibre assay

Cells were exposed to either DMSO or 500 μM uridine for 24 h, pulse-labelled with 25 μM CldU for 30 min, washed with medium and pulse-labelled with 25 μM IdU for U2OS and hTERT-RPE1 cells or 250 μM IdU for MCF7 cells for 30 min. Labelled cells were harvested and DNA fibre spreads were prepared by gravitational flow on microscopy slides as described elsewhere [42]. CldU was detected by incubating acid-treated fibre spreads with rat anti-BrdU monoclonal antibody (AbD Serotec; cat# MCA2060; and Abcam; ab6326), whereas IdU was detected using mouse anti-BrdU monoclonal antibody (BD Biosciences; cat# 347580) for 1 h at 37 °C. Slides were fixed with 4% PFA and incubated with goat anti-rat Alexa Fluor 555 or goat anti-mouse Alexa Fluor 488 for 1–2 h at 4 °C. Fibres were examined using a Zeiss (Jena, Germany) LSM780 or LSM980 Airyscan2 confocal laser scanning microscope with a 63x oil immersion objective. For quantification of replication structures, at least 250 structures were counted per experiment. The lengths of red (AF 555) and green (AF 488)-labelled patches were measured using the ImageJ software (National Institutes of Health; http://rsbweb.nih.gov/ij/) and arbitrary length values were converted into micrometres using the scale bars created by the microscope.

### EdU incorporation

U2OS and hTERT-RPE1 cells were treated with 50 μM pyrazofurin for 4 days, and MCF7 cells were treated with 0.1 μM pyrazofurin for 4 days in 96-well plates. EdU (10 μM) was added for 20 min. Cells were fixed in 4% PFA and permeabilized in 0.3% Triton X-100. The click reaction was performed as follows: 859 μl PBS, 40 μl CuS04 (100 mM), 1 μl Atto 488 (Sigma-Aldrich), and 100 μl 100 mM ascorbic acid for 30 min at RT. Cells were then washed and imaged at the ImageXpress XLS (Molecular Devices) or Celldiscoverer7 (Zeiss). Data were analyzed with CellProfiler-3.0.0 or Zen Blue (Zeiss).

### Statistical analysis

Unpaired two-tailed Student’s *t* test was used for comparisons between two groups. When the data points did not follow a normal distribution, a two-tailed Mann–Whitney test was performed. All statistical analyses were performed using GraphPad Prism. All experiments were repeated at least 3 times.

### LC-MS nucleotide measurements

Measurements were carried out at Creative Proteomics (Shirley, NY, USA). To each cell pellet 100 μL of water was added. Cells were lysed on a MM 400 mill mixer with the aid of two metal balls at a shaking frequency of 30 Hz for 1 min. Then, 400 μL of methanol was then added to each tube and the samples were homogenized again for 1 min twice, followed by sonication for 1 min in an ice-water bath. The samples were placed at −20 °C for 30 min before centrifugal clarification at 21,000 × *g* and 5 °C for 5 min. The clear supernatants were collected. The protein pellets were used for the protein assay using the standard Bradford procedure. Serially diluted, mixed standard solutions of dNTPs were prepared at the concentrations of 0.0002 to 10 μM in an internal standard solution containing 13 C or D-labelled ATP, GTP and UTP. Twenty microliters of the supernatant of each sample solution were mixed with 180 μL of the same internal standard solution. Ten-microliter aliquots of the sample solutions and the standard solutions were injected onto a C18 UPLC column (2.1*100 mm, 1.8 μm) for UPLC-MRM/MS runs with (-) ion detection on a Waters Acquity UPLC system coupled to a Sciex QTRAP 6500 Plus MS instrument, with the use of a tributylamine formate solution (solvent A) and acetonitrile (solvent B) as the mobile phase for gradient elution with an efficient gradient of 5% to 50% B in 20 min at 0.30 mL/min and 50 °C. Concentrations of the detected analytes were calculated with internal standard calibration by interpolating the constructed linear regression curves of individual compounds, with the analyte-to-internal standard peak area ratios measured from the sample solutions in each assay.

### In vivo studies

Ten-week-old female NOD/SCID wild-type mice (Charles River, Kent, UK) kept on a 12-h/12-h light/dark cycle with free access to food and water were used in this study, and the study was carried out in accordance with local guidelines and with Home Office approval under project licence PP9172663, University of Sheffield, UK. MCF7-GFP-Luc cells were generated by infection with LVP020 (Amsbio) and selected with 1 μg/ml puromycin.

On day 0 800.000 cells in 20 μl Media, 10% Matrigel were injected on either side intra nipple (4&9). The drinking water was supplemented with 4 mg/L 17-β-estradiol. Tumour growth was monitored in live mice using an IVIS Lumina II system (Caliper Life Sciences, UK). Here, 30 mg/kg of D-Luciferin (Invitrogen, UK) was injected s.c. 5 min before imaging. Mice were imaged by ventral exposure. Images were analyzed in Living Image software by creating a region of interest (ROI) around the tumour(s); luminescence signal was acquired in radiance (photons/second).

### Cell fractionation

Cell pellets were collected and lysed according to [43] to separate the soluble (cytosolic) and insoluble (nuclei, membranes) fraction. Buffer composition for soluble fraction is: 42 μg/ml Digitonin, 2 mM MgCl_2_, 150 mM NaCl, 0.2 mM EDTA, 20 mM HEPES, pH 7.4. Insoluble fraction was lysed in RIPA buffer.

### Cell cycle analysis

Cells were seeded on 10 cm cell culture petri dishes and cell cycle synchronisation was achieved using the double thymidine block technique [44]. Cells were arrested at the G1/S boundary twice with 2 mM thymidine and samples were taken at the indicated time points after thymidine release. CCNB1 antibody was used to confirm cell cycle phases.

### Database analysis

Data was retrieved from cBioPortal Breast CancerPanel excluding duplicate TCGA studies. Altered group is defined as patients with at least one alteration in the *NUDT22* gene and unaltered group with no alterations in the *NUDT22* gene.

Overall Survival (OS) data for the Kaplan Meier plots was retrieved from Breast RNA-seq [45]. Gene expression data was retrieved from UCSC Xenabrowser comparing datasets from TCGA matched versus GTEx normal tissue or CCLE data.

## Supplementary information

Supplementary Fig. 1

Supplementary Fig. 2

Supplementary Fig. 3

Supplementary Fig. 4

Supplementary Fig. 5

Supplementary Fig. 6

Supplementary Fig. 7

Supplemental Fig. 8

Supplementary Figure legends

## Data Availability

The datasets generated during and/or analysed during the current study are available from the corresponding author on reasonable request or here: 10.6084/m9.figshare.19823347.
