# Supplementary figures and images for: NUDT22 promotes cancer growth through pyrimidine salvage
Source: Oncogene. 2023 Mar 4;42(16):1282–93. doi: 10.1038/s41388-023-02643-4 (PMC10101856; doi:10.1038/s41388-023-02643-4)

# Supplementary Fig. 1

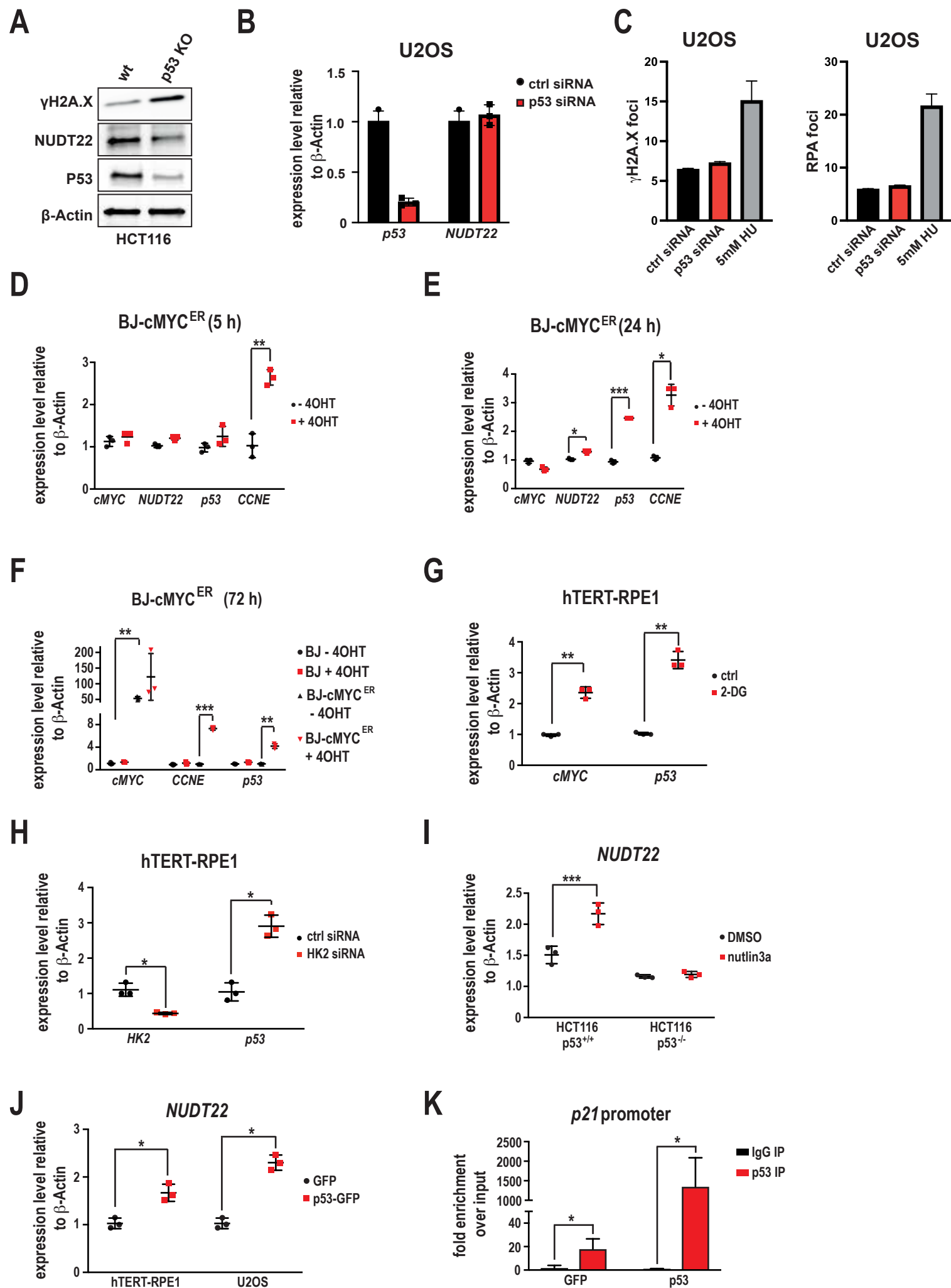

Supplement: Supplementary file 1 — Supplementary Fig. 1 [file 41388_2023_2643_MOESM1_ESM.pdf]

# Supplementary Fig. 2

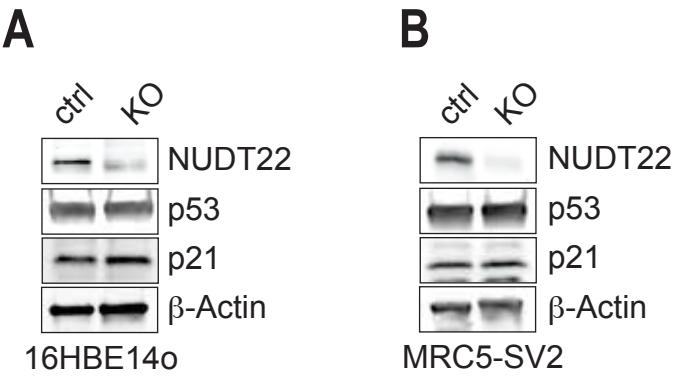

Supplement: Supplementary file 2 — Supplementary Fig. 2 [file 41388_2023_2643_MOESM2_ESM.pdf]

# Supplementary Fig. 3

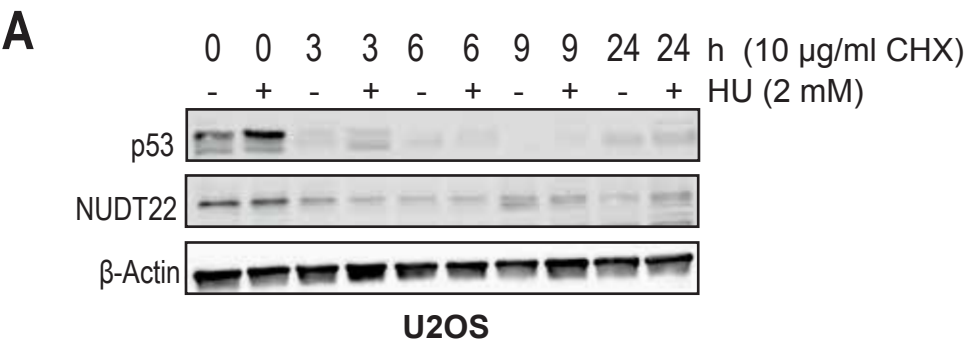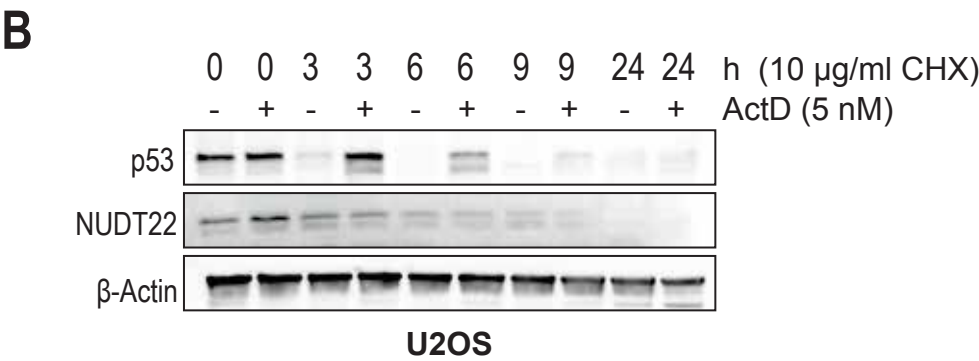

Supplement: Supplementary file 3 — Supplementary Fig. 3 [file 41388_2023_2643_MOESM3_ESM.pdf]

# Supplementary Fig. 4

A

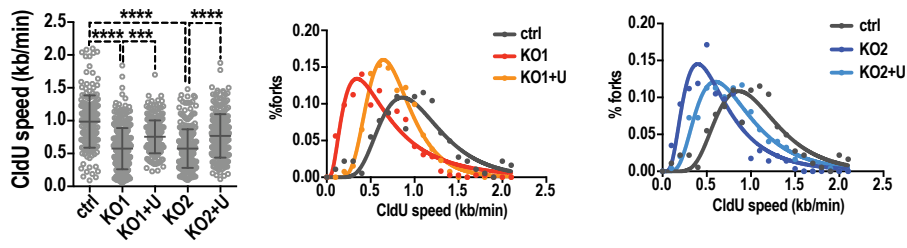

B

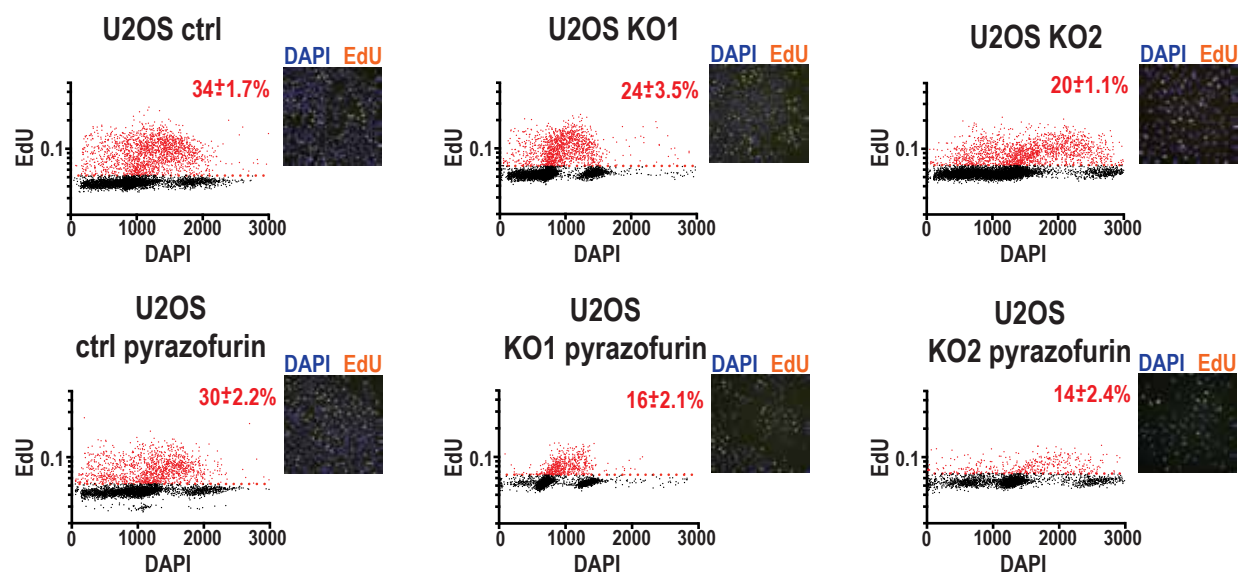

C

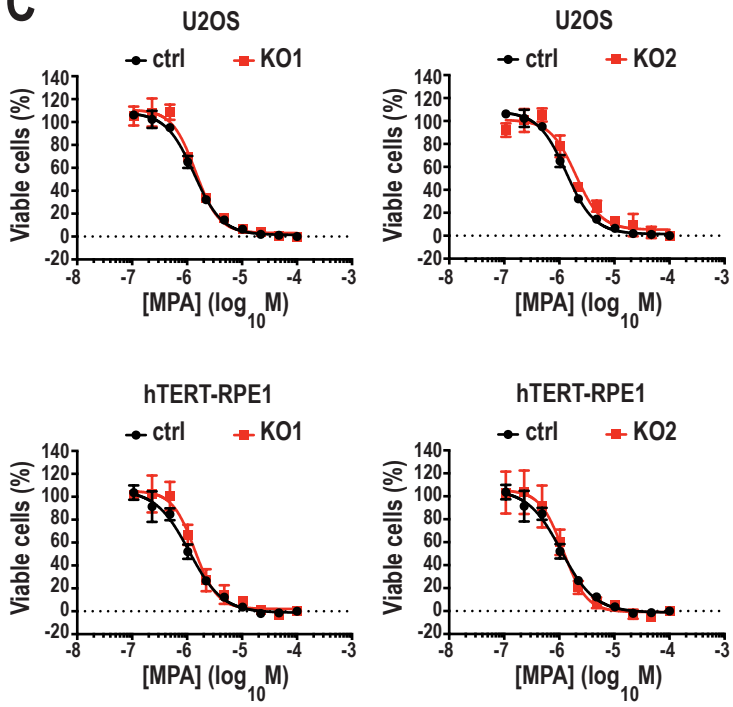

D

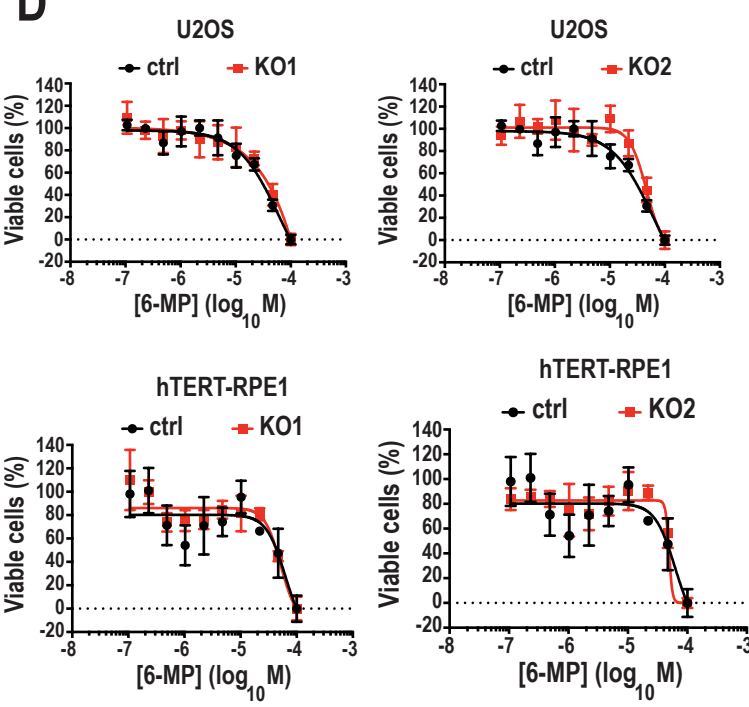

Supplement: Supplementary file 4 — Supplementary Fig. 4 [file 41388_2023_2643_MOESM4_ESM.pdf]

# Supplementary Fig. 5

A

16HBE14o

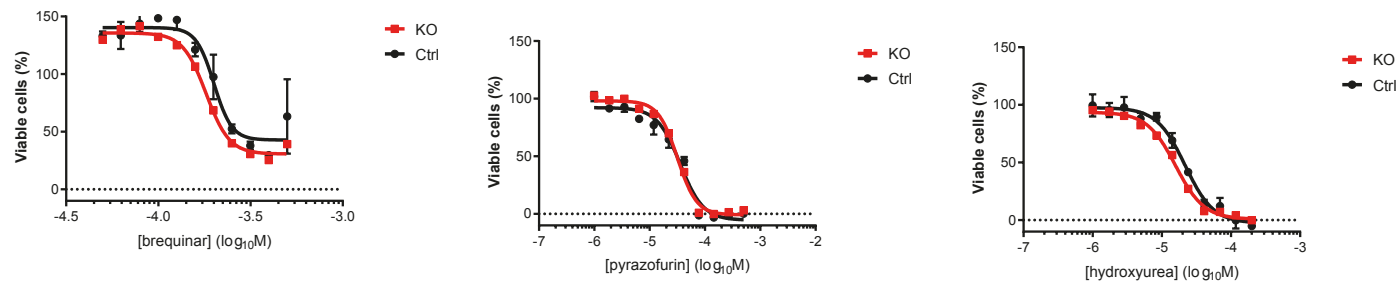

MRC5-SV2

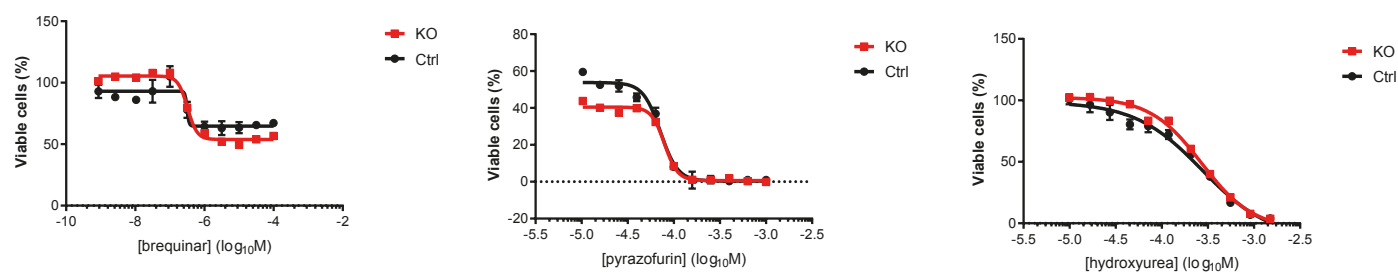

B

16HBE14o

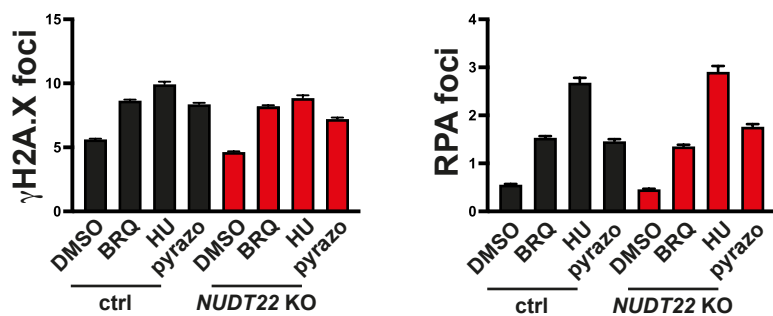

MRC5-SV2

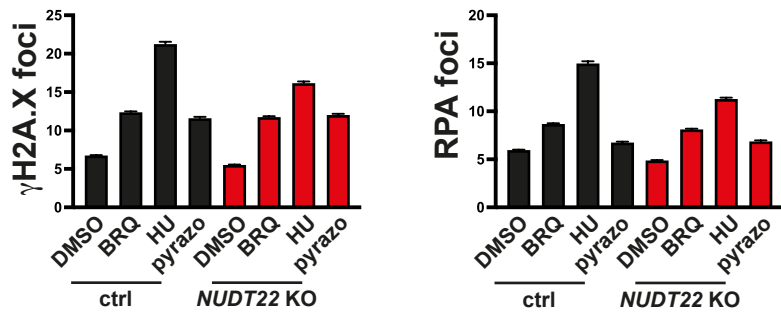

Supplement: Supplementary file 5 — Supplementary Fig. 5 [file 41388_2023_2643_MOESM5_ESM.pdf]

# Supplementary Fig. 6

A

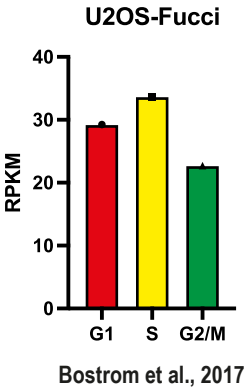

B

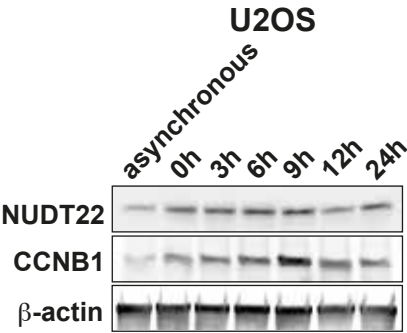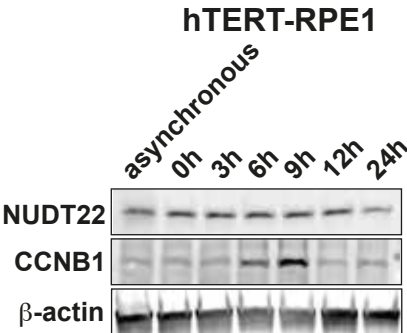

Supplement: Supplementary file 6 — Supplementary Fig. 6 [file 41388_2023_2643_MOESM6_ESM.pdf]

# Supplementary Fig. 7

A

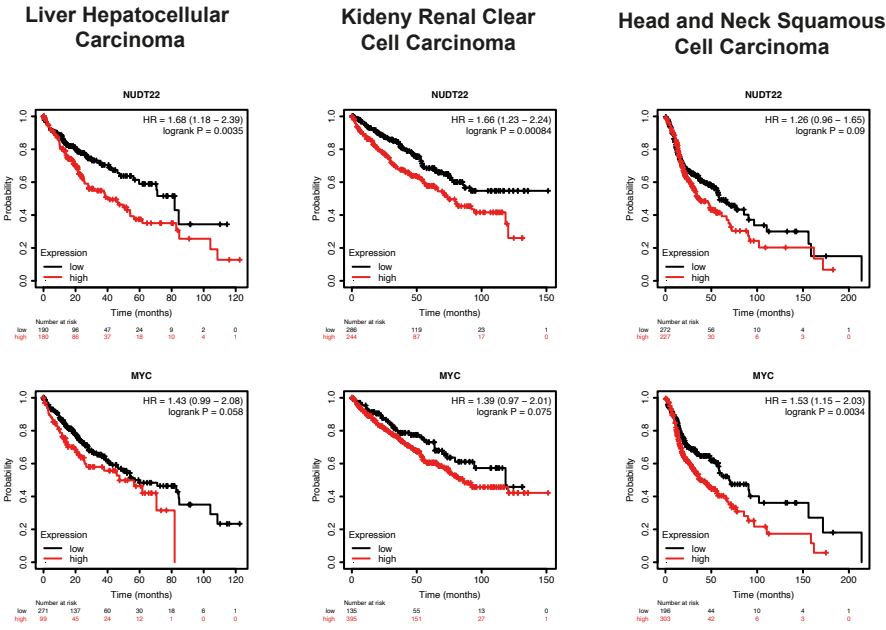

B

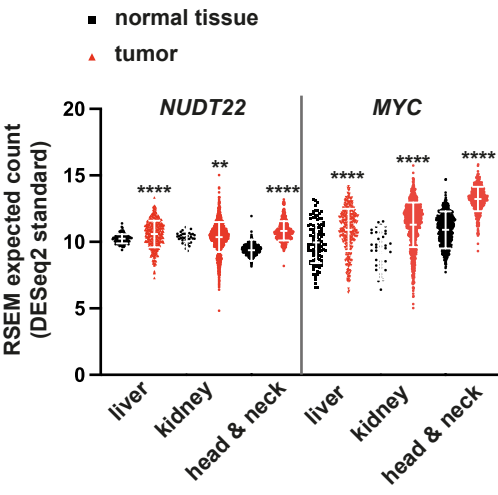

C

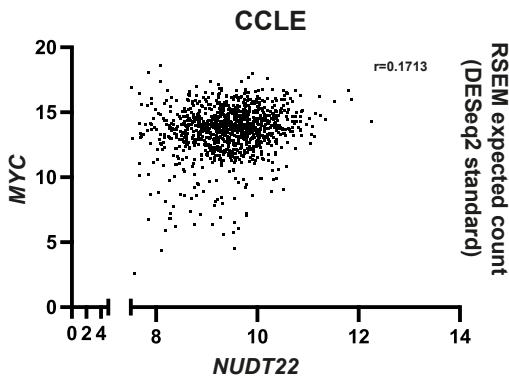

D

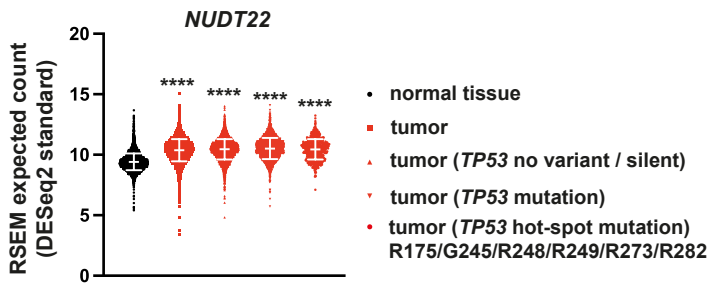

E

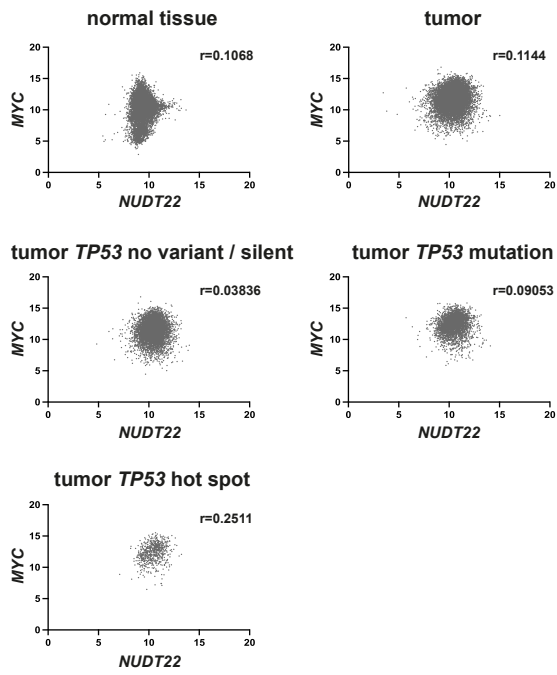

Supplement: Supplementary file 7 — Supplementary Fig. 7 [file 41388_2023_2643_MOESM7_ESM.pdf]

# Supplementary Fig. 8

A

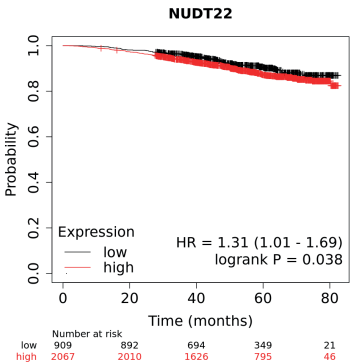

B

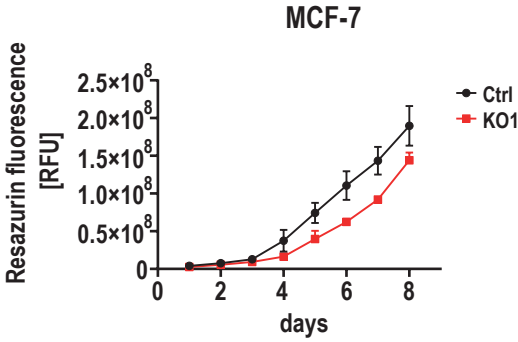

C

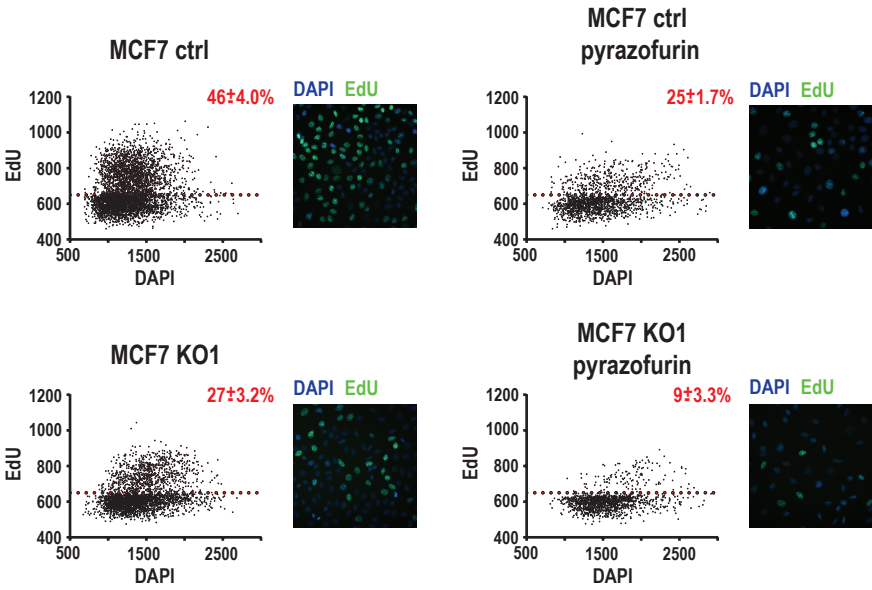

D

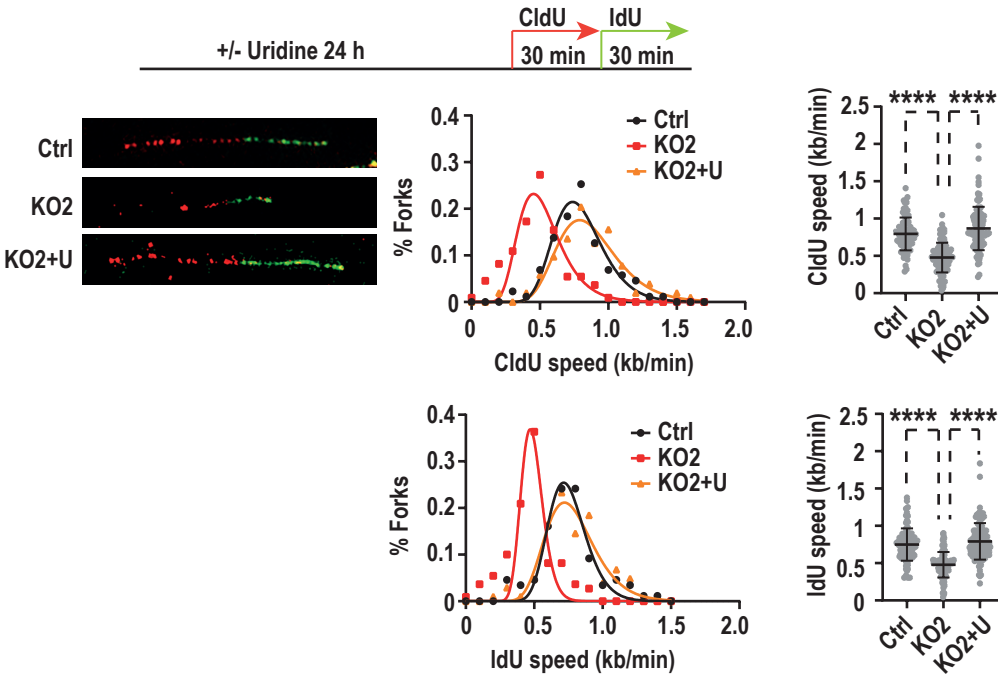

Supplement: Supplementary file 8 — Supplemental Fig. 8 [file 41388_2023_2643_MOESM8_ESM.pdf]
