# Supplementary material for: NUDT22 promotes cancer growth through pyrimidine salvage
Source: Oncogene. 2023 Mar 4;42(16):1282–93. doi: 10.1038/s41388-023-02643-4 (PMC10101856; doi:10.1038/s41388-023-02643-4)
Supplement: Supplementary file 9 — Supplementary Figure legends [file 41388_2023_2643_MOESM9_ESM.docx]

**Supplementary Fig. 1**

**A** Reduced NUDT22 protein levels and increased γH2A.X protein levels in HCT116 p53 KO cells. **B** qRT-PCR analysis of NUDT22 gene expression after siRNA mediated p53 silencing in U2OS cells reveals no effect on *NUDT22* expression levels. **C** Microscopy assisted DNA damage foci analysis after siRNA mediated p53 silencing in U2OS cells. **D** Short-term activation of cMYC induced expression of the direct target gene *CCNE* (P=0.0089) but not of *NUDT22* or *p53.* **E** Increased expression of both *NUDT22* and *p53* after cMYC^ER^ activation with tamoxifen (4-OHT) for 24 h (*NUDT22* P=0.038; *p53* P=0.0006; *CCNE* P=0.01). **F** Activation of cMYC^ER^ (4-OHT) for 72 h increased the expression of *CCNE* and *p53* (*cMYC* P=0.0041; *CCNE* P=0.0007; *p53* P=0.0047). **G** The exposure of hTERT-RPE1 cells to 2-DG for 48 h induced *cMYC* and *p53* expression (*cMYC* P=0.0037; *p53* P=0.0033). **H** Increased *p53* expression after *HK2^siRNA^* transfection (*HK2* P=0.0314; *p53* P=0.0234). **I** Nutlin3a exposure induced *NUDT22* expression only in the *p53^+/+^* HCT116 cell line and not in the isogenic *p53^-/-^* HCT116 cell line (*NUDT22* P=0.001). **J** Increased *NUDT22* expression after *p53* overexpression (hTERT-RPE1 P=0.0314; U2OS P=0.0144). **K** qPCR for the *P21* promoter after ChIP with a p53(DO1) antibody. GFP served as a transfection control, (*P21* P=0.034 and 0.033). P-values were calculated by paired t-test. Data are presented as the mean values with standard deviation.

**Supplementary Fig. 2**

Western blot analysis of p53 and p21 protein levels in **A** 16HBE14o *NUDT22* knockout cells and **B** MRC5-SV2 *NUDT22* knockout cells.

**Supplementary Fig. 3**

**A** NUDT22 protein is slightly increased following 2mM HU exposure. Translation inhibition with CHX reveals rapid degradation of NUDT22 and p53 protein. **B** NUDT22 protein is slightly increased following 5nM ActD exposure. Translation inhibition with CHX reveals rapid degradation of NUDT22 while p53 protein levels are maintained for a longer period of time.

**Supplementary Fig. 4**

**A** Replication fork speed (CldU incorporation) in U2OS ctrl and *NUDT22* KO cells. **B** Quantification of the percentage of EdU-positive cells (ctrl::1-2 P=0.0109; ctrl::3-6 P=0.0003; 1-2 DMSO::1-2 pyrazofurin P=0.0227; 3-6 DMSO::3-6 pyrazofurin P=0.0109)|. P-values were calculated by unpaired t-test. Errors as the mean with SD) and representative images. **C** Dose response curves of ctrl and *NUDT22* KO U2OS and hTERT-RPE1 cells exposed to MPA and **D** 6-MP for 96 h.

**Supplementary Fig. 5**

**A** Dose response experiment of 16HBE14o and MRC5-SV2 *NUDT22* KO cells after 4 days of brequinar, pyrazofurin or hydroxyurea exposure. **B** 16HBE14o and MRC5-SV2 *NUDT22* KO cells do not have increased number of DNA damage foci (γH2A.X, RPA) over respective control cells after brequinar, pyrazofurin or hydroxyurea exposure for 4 days.

**Supplementary Fig. 6**

**A** Gene expression analysis of *NUDT22* during the cell cycle (data from Boström et al., 2017). **B** Double thymidine block cell cycle synchronization analysis of NUDT22 protein abundance across the cell cycle in U2OS and hTERT-RPE1 cells.

**Supplementary Fig. 7**

**A** Kaplan Meier overall survival (OS) plots or RNAseq data. High expression of *NUDT22* and *cMYC* correlates with worse survival outcome in patients with liver hepatocellular carcinoma, kidney renal clear cell carcinoma and head and neck squamous cell carcinoma. Especially liver cancer has been shown to frequently carry cMYC driver mutations (Shachaf et al., 2005 Nature: MYC inactivation uncovers pluripotent differentiation and tumor dormancy in hepatocellular cancer). **B** RSEM expected count expression analysis of *NUDT22* and *cMYC* in liver, kidney and head and neck cancer versus respective normal tissue confirms significantly increased expression levels (P-values calculated by Mann-Whitney test). **C** Correlation of gene expression of *NUDT22* and *MYC.* Data retrieved from CCLE. **D** RSEM expected count expression analysis of *NUDT22* in cancer with wildtype p53 (TP53 no variant/silent), p53 mutations or p53 hot-spot mutations (P-values calculated by Mann-Whitney test). **E** Correlation of gene expression of *NUDT22* and *MYC* in tumors grouped based on their *p53* mutational status.

**Supplemental Fig. 8**

**A** Kaplan Meier plot of overall survival OS of breast cancer patients (Breast RNA-seq) grouped based on high and low *NUDT22* expression. **B** MCF7 *NUDT22* KO cells grew slower than their parental control cells. **C** *NUDT22* KO MCF7 cells have reduced EdU incorporation, which is further reduced by pyrazofurin exposure **D** Reduced replication fork speed in *NUDT22* KO2 MCF7 cells can be rescued by uridine supplementation.
